# Supplementary figures and images for: Land use and land cover dynamics and traditional agroforestry practices in Wonchi District, Ethiopia
Source: PeerJ. 2022 Feb 22;10:e12898. doi: 10.7717/peerj.12898 (PMC8877395; doi:10.7717/peerj.12898)

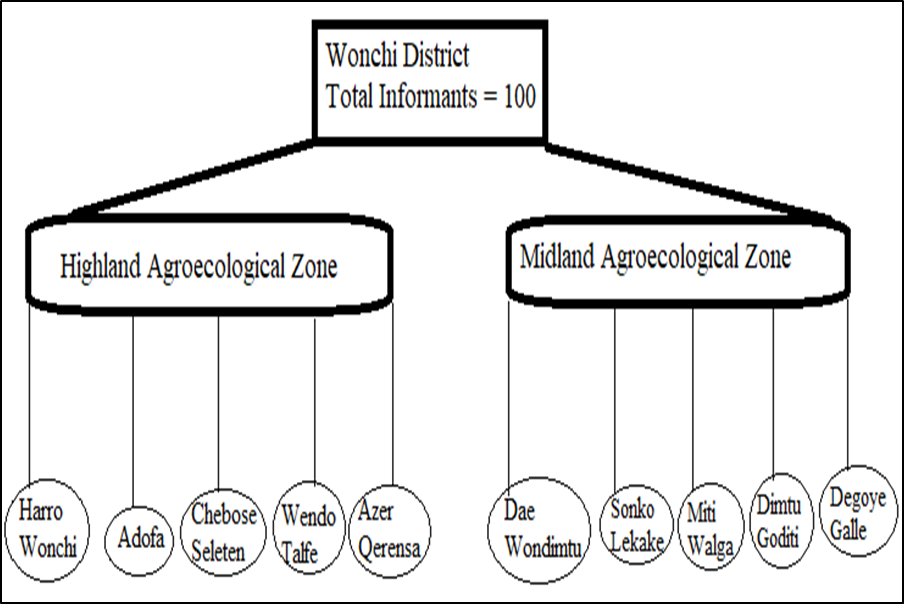

Supplement: Supplemental Information 1 [file peerj-10-12898-s001.png]
