# Supplementary material for: Land use and land cover dynamics and traditional agroforestry practices in Wonchi District, Ethiopia
Source: PeerJ. 2022 Feb 22;10:e12898. doi: 10.7717/peerj.12898 (PMC8877395; doi:10.7717/peerj.12898)
Supplement: Supplemental Information 2 — raw data [file peerj-10-12898-s002.docx]

| Demographic Variables | Gender of informants | | Total (N = 100) |
| --- | --- | --- | --- |
| Age groups (in years) | Male | Female | Frequency |
| 30–39 | 26 | 21 | 47 |
| 40–59 | 20 | 17 | 37 |
| ≥60 | 14 | 2 | 16 |
| Total | 60 | 40 | 100 |
| Educational status/grades | Male | Female | Frequency |
| Uneducated | 13 | 22 | 35 |
| 1–6 | 26 | 14 | 40 |
| 7–12 | 17 | 3 | 20 |
| >12 | 4 | 1 | 5 |
| Total | 60 | 40 | 100 |
