# Supplementary material for: Land use and land cover dynamics and traditional agroforestry practices in Wonchi District, Ethiopia
Source: PeerJ. 2022 Feb 22;10:e12898. doi: 10.7717/peerj.12898 (PMC8877395; doi:10.7717/peerj.12898)
Supplement: Supplemental Information 3 — raw data [file peerj-10-12898-s003.docx]

| LULC type | | Description |
| --- | --- | --- |
| Agroforestry cover |  | Enset (*Ensete ventricosum*) and perennial fruit tree dominated homegarden, vegetables, and croplands with scattered trees grown, used for various purposes such as, keeping soil fertility, shading, bee hives, traditional medicine, food, fencing, and construction grown in farmland and homegarden. |
| Cropland |  | Includes areas used for annual and seasonal crop production and fallow lands/pastures. The annual crops include barely (*Hordeum vulgare*) in the highlands and teff (*Eragrostis tef*) and maize (*Zea mays*) in the midlands. |
| Forest cover |  | Areas covered with dense trees forming closed or nearly closed  canopies. It includes plantation of *Eucalyptus* spp.*, Cupressus* sp., and *Juniperus procera* and *Hagenia abyssinica, Olea europaea* subsp. *cuspidata* and *Podocarpus falcatus* in natural forests. |
| Settlement and roads |  | Includes buildings in towns as well as rural areas, and roads. Degraded and bare land that has little or no vegetation cover such as exposed rocks, churches, mosques, health centers, and schools are included in this land-use type. |
| Shrub cover |  | Areas where are shrubs the dominant woody vegetation (less than 2 m tall) cover with small trees, grasses in wetlands, and rehabilitation areas. |
| Water body |  | Includes all water body systems such as streams, rivers, lakes, ponds, and swamps. |
