# Supplementary material for: Land use and land cover dynamics and traditional agroforestry practices in Wonchi District, Ethiopia
Source: PeerJ. 2022 Feb 22;10:e12898. doi: 10.7717/peerj.12898 (PMC8877395; doi:10.7717/peerj.12898)
Supplement: Supplemental Information 4 — raw data: Habitat: Fp = Forest patch; Gl = Grazing land; Hg = Homegarden; Cl = Crop land; Species origin to Ethiopia: Ig = Indigenous; Id = Introduced; Ed = Endemic to the flora area; Sites (Kebeles): Highland (HW = Harro Wonchi, Ad = Adofa, CS = Chebose Seleten , WT = Wendo Talfe, AQ = Azer Qerensa); Midland (DW = Dae Wandimtu , SL = Sonko Lekake, MW = Miti Walga, DGo = Dimtu Goditi, DG = Degoye Galle). a Stands for species that are restricted to only in the midland agro-ecological zone and b stands for species that are confined to only in highland agro-ecological zone. [file peerj-10-12898-s004.docx]

| **S. No.** | **Species name** | **Family** | **Habitat** | **GPS72H Location** | **Site** | **Main use** | **Origin** | **Plot No.** |
| --- | --- | --- | --- | --- | --- | --- | --- | --- |
| 1 | *Agarista salicifolia ^b^* (Comm. ex Lam.) Don | Ericaceae | Fp, Gl | 3290 m, 8.802135 N, 37.862449 E | HW, CS, AQ | Construction, firewood, leaf parts used as a local medicine for wounds | Ig | 002 |
| 2 | *Agave sisalana* Perro ex Eng. | Agavaceae | Gl, Hg | 2303 m, 8.65293 N, 37.954932 E | DW | Live fence, firewood when dry, hollow-wood used for construction of beehives, flowers attract honeybees, | Ig | 036 |
| 3 | *Albizia schimperiana* Oliv. | Fabaceae | Fp, Gl, Hg | 2087 m, 8.591932 N, 37.930255 E | DW, SL, MW, DGO, DG | Live shade, control erosion, firewood, construction, furniture | Ig | 047 |
| 4 | *Allium cepa* L. | Alliaceae | Cl, Hg | 2267 m, 8.65356 N, 37.907198 E | All | A very important vegetable and spice in the preparation of pepper, | Id | 047 |
| 5 | *Allium sativum* L. | Alliaceae | Cl, Hg | 2896 m, 8.754183 N, 37.913359 E | CS, AQ, DGo, DG | A very important vegetable, spice, and nutraceutical food. | Id |  |
| 6 | *Arundinaria alpina ^b^* K. Schum. | Poaceae | All | 3290 m, 8.802136 N, 37.86251 E | HW, Ad, CS, AQ | Construction, house furniture, basketry, walking sticks timber available in the market, maintain streams, fences | Ig | 001 |
| 7 | *Bambusa polymorpha* Munro. | Poaceae | Cl, Gl, Hg | 2108 m, 8.60025 N, 37.881603 E | MW, DG | Protecting the soil from heavy erosion, fodder for domestic animals | Id | 057 |
| 8 | *Bersama abyssinica* Fresen | Melianthaceae | Gl, Hg | 2103 m, 8.35913 N, 37.57252 E | MW, DG | Shade for coffee, street tree, control erosion, firewood | Ig | 054 |
| 9 | *Beta vulgaris* var. *esculenta*^a^ L. | *Chenopodiaceae* | Hg | 1997 m, 8.58315 N, 37.84197 E | DW, MW, DG | Vegetable, the roots are sliced and used as stew | Id | 054 |
| 10 | *Brassica carinata* A. Br. | *Brassicaceae* | Hg | 3151 m, 8.00664 N, 37.863707 E | HW, Ad, CS, WT, AQ | The leaves are eaten cooked as a vegetable | Ig | 006 |
| 11 | *Brassica oleracea*^a^ L. | *Brassicaceae* | Hg | 2087 m, 8.591932 N, 37.930255 E | MW | The leaves are eaten cooked as a vegetable | Ig | 059 |
| 12 | *Brucea antidysenterica* J. F. Mill. | Simaroubaceae | Fp, Gl | 2308 m, 8.655327 N, 37.95476 E | DW, SL | Different parts have been used for treating various disease including diarrhea, rabies, wounds, and abdominal pains, control soil erosion | Ig | 036 |
| 13 | *Buddleja polystachya* Fresen. | Loganiaceae | Fp, Gl, Hg | 2305 m, 8.656886 N, 37.954046 E | DW | Construction, firewood, live fence, flowers provide pollen and nectar for honeybees, pounded leaves used as local medicine against wound and skin disease | Ig | 035 |
| 14 | *Calpurnia aurea* (Ait.) Benth. | Fabaceae | Fp, Gl, Hg | 2311 m, 8.652808 N, 37.9045541 E | SL, DGo, DG | Live fence, farm tools, firewood, seeds used as a cure for dysentery, control erosion | Ig | 049 |
| 15 | *Capsicum annuum* L. | *Solanaceae* | Hg | 2305 m, 8.656886 N, 37.954046 E | DW | Vegetable and spices | Id | 058 |
| 16 | *Capsicum* sp. | *Solanceae* | Hg | 2267 m, 8.65356 N, 37.907198 E | All | Vegetable, sweet pepper (Abasoya) used as nutraceutical food | Id | 047 |
| 17 | *Carica papaya*^a^ L. | Caricaceae | Cl, Hg | 1997 m, 8.58315 N, 37.84197 E | DGo, DG | Tree crop, ripe edible fruit, making juice, treating gastritis | Id | 054 |
| 18 | *Carissa spinarum* L. | Apocynaceae | Fp, Gl, Hg | 2311 m, 8.652808 N, 37.9045541 E | WT, SL | Live fence, leaves are forage for goats and sheep, edible fruit, firewood | Ig | 049 |
| 19 | *Casimiroa edulis* La Llave | Rutaceae | Hg | 1997 m, 8.58315 N, 37.84197 E | DGo | Tree crop, ripe edible fruit | Id | 054 |
| 20 | *Catha edulis*^a^ (Vahl) Forssk. ex Endl. | Celastraceae | Cl, Hg | 1997 m, 8.58315 N, 37.84197 E | DW | Tree crop whose shoots and leaves are used for commercial purposes | Ig | 054 |
| 21 | *Cicer arietinum* L. | Fabaceae | Cl, Hg | 2305 m, 8.656886 N, 37.954046 E | All | Legume crop that can be added to various traditional dishes like stew, soup | Id | 060 |
| 22 | *Citrus limon*^a^ (L.) Burm.f. | Rutaceae | Cl, Hg | 1997 m, 8.58315 N, 37.84197 E | DW, MW | Tree crop, fruits used as a source of vitamins and for sale in local markets | Id | 054 |
| 23 | *Citrus sinensis*^a^ (L.) Osb. | Rutaceae | Cl, Hg | 1997 m, 8.58315 N, 37.84197 E | DW, MW | Tree crop, fruits used as a source of vitamins and for sale in local markets | Id | 054 |
| 24 | *Coffea arabica*^a^ L. | Rubiaceae | Cl, Hg | 1997 m, 8.58315 N, 37.84197 E | DW | Tree crop whose beans used for commercial purposes | Ig | 054 |
| 25 | *Conyza hypoleuca ^b^* A. Rich. | Asteraceae | Fp, Gl | 3042 m, 8.783696 N, 37.838218 E | HW, Ad | Firewood, erosion control in sloped area | Ig | 020 |
| 26 | *Cordia africana*^a^ Lam. | Boraginaceae | All | 2308 m, 8.655327 N, 37.95476 E | DW, SL, DGo, DG | Timber trees, wood used for making beehives, drums, furniture, flowers used as forage for honeybees, edible fruits, control erosion | Ig | 036 |
| 27 | *Croton macrostachyus*^a^ Del. | Euphorbiaceae | All | 2309 m, 8.655317 N, 37.954756 E | DW, SL, MW, DGo, DG | Construction, furniture, firewood, shade for cattle, watery droplet at the fresh shoot used as local medicine to stop bleeding | Ig | 036 |
| 28 | *Cuminum cyminum*^a^ L. | Apiaceae | Hg | 2087 m, 8.591932 N, 37.930255 E | MW | Spice, fruits are sold in markets and used in the preparation of pepper | Id | 058 |
| 29 | *Cupressus lusitanica* Mill. | Cupressaceae | Fp, GL, Hg | 3073 m, 8^.^469280 N, 37^.^52558 E | HW, WT, DW | Plants as a timber tree for marketing, construction, live fences | Ig | 003 |
| 30 | *Daucus carota*^a^ L. | *Apiaceae* | Hg | 1997 m, 8.58315 N, 37.84197 E | AQ, MW | Vegetable, used in raw form as a salad or cooked, steamed or fried, and served with various sauces | Id | 054 |
| 31 | *Dendrocalamus asper* (Schult. & Schult.f.) Backer ex K.Heyne. | Poaceae | Cl, Gl, Hg | 2108 m, 8.60025 N, 37.881603 E | MW, DG | Protecting the soil from heavy erosion, fodder for domestic animals | Id | 057 |
| 32 | *Dendrocalamus barbatus* Hsueh et D. Z. Li. | Poaceae | Cl, Gl, Hg | 2108 m, 8.60025 N, 37.881603 E | MW, DG | Protecting the soil from heavy erosion, fodder for domestic animals | Id | 057 |
| 33 | *Discopodium penninervium* Hochst. | Solanaceae | Fp, GL, Hg | 2896 m, 8.754183 N, 37.913359 E | Ad, CS, AQ | Live fence, control soil erosion | Ig | 032 |
| 34 | *Dovyalis abyssinica* (A. Rich.) Warb. | Flacourtiaceae | Fp, Gl, Hg | 2108 m, 8.60025 N, 37.881603 E | AQ, MW, DG | Live and dried fences, windbreaks, protecting the soil from heavy erosion, firewood | Ig | 058 |
| 35 | *Echinops longisetus ^b^* A.Rich. | Asteraceae | Fp, Gl | 3151 m, 8.00664 N, 37.863707 E | HW, AQ | Fodder, flowers provide pollen and nectar for honeybees, local medicine against headache | Ed | 005 |
| 36 | *Ekebergia capensis* Sparrm. | Meliaceae | Fp, Hg | 2103 m, 8.35913 N, 37.57252 E | MW, DG | Shade for coffee, street tree, control erosion, firewood | Ig | 054 |
| 37 | *Ensete ventricosum* (Welw.) Cheesman) | Musaceae | Cl, Hg | 2911 m, 8.754148 N, 37.913583 E | All | All parts are very important as food in many forms, provide fibers for rope, leaves for wrapping bread, and others | Ig | 029 |
| 38 | *Eragrostis tef* (Zucc.) | Poaceae | Cl, Hg | 2305 m, 8. N, 37.954046 E | All | Cereal crop used for making injera, a special Ethiopian bread | Ig | 023 |
| 39 | *Erica arborea ^b^* L. | Ericaceae | Fp, Gl | 3215 m, 8.803247 N, 37.865236 E | HW, Ad, CS, AQ | Construction, firewood, control soil erosion | Ig | 004 |
| 40 | *Erythrina brucei* Schweinf. | Fabaceae | All | 2108 m, 8.60025 N, 37.881603 E | WT, DG | Maintaining soil fertility, woods for the construction of beehives, flowers provide pollen and nectar for honeybees | Ed | 055 |
| 41 | *Eucalyptus globulus* Labill. | Myrtaceae | Fp, GL, Hg | 3290 m, 8.802136 N, 37.86251 E | All | Timber available in market, construction, firewood, live fence, leaf parts used as a local medicine for the common cold | Id | 001 |
| 42 | *Euphorbia tirucalli*^a^ L. | Euphorbiaceae | Hg | 2108 m, 8.60025 N, 37.881603 E | DGo | Live fence, control soil erosion | Ig | 055 |
| 43 | *Ficus sur*^a^ Forssk | Moraceae | Cl, Fp, Gl | 2308 m, 8.655327 N, 37.95476 E | CS, WT, DW | Used for protecting soil from erosion, shade for cattle, part of riparian vegetation, maintain streams | Ig | 035 |
| 44 | *Ficus vasta*^a^ Forssk | Moraceae | Cl, Fp, Gl | 2308 m, 8.655327 N, 37.95476 E | DW, MW, DGo | Used for protecting soil from erosion, shade for cattle, part of riparian vegetation, maintain streams | Ig | 035 |
| 45 | *Grevillea robusta* R. Br. | Proteaceae | Fp, Gl, Hg | 2267 m, 8.65356 N, 37.907198 E | SL | Construction, timber, furniture, control erosion, firewood | Ig | 051 |
| 46 | *Guizotia abyssinica***^a^** (L.f.) Cass. | Asteraceae | Cl, Hg | 2087 m, 8.591932 N, 37.930255 E | MW | Oilseed yields a high-quality edible oil; flowers provide pollen and nectar for honeybees | Ig | 057 |
| 47 | *Hagenia abyssinica^b^* (Bruce) Gmel | Rosaceae | All | 3290 m, 8.802136 N, 37.86251 E | HW, Ad, CS, WT, AQ | Timber for the market, construction, furniture, soil fertility, female flowers used as locally traded medicine for tapeworm infestation | Ig | 001 |
| 48 | *Hordeum vulgare* L. | Poaceae | Cl, Hg | 2911 m, 8.754148 N, 37.913583 E | All | Cereal crop with many forms/varieties used for making food, local beverage, and roasted grain and chaffs as forage for cattle | Ig | 054 |
| 49 | *Hypericum revolutum ^b^* Vahl | Hypericaceae | All | 3151 m, 8.00664 N, 37.863707 E | HW, Ad | Firewood, fodder, roots used as a local medicine for stomach problem | Ig | 006 |
| 50 | *Ilex mitis ^b^* (L.) Radlk. | Aquifoliaceae | Fp, Gl | 3029 m, 8.784141 N, 37.837693 E | Ad | Construction, firewood, farm tools | Ig | 019 |
| 51 | *Inula confertiflora ^b^* A. Rich. | Asteraceae | Fp, Gl | 3042 m, 8.783696 N, 37.838218 E | Ad, AQ | Dried parts used for humans during child birth in the form of fumigation and pounded leaves for treating eyes of cattle | Ed | 020 |
| 52 | *Jacaranda mimosifolia* D. Don | Bignoniaceae | Gl, Hg | 2087 m, 8.591932 N, 37.930255 E | DW | Shade for coffee, street tree, control erosion, firewood | Ig | 054 |
| 53 | *Juniperus procera* Hochst. ex Endl. | Cupressaceae | All | 3151 m, 8.00664 N, 37.863707 E | HW, Ad, CS, AQ, WT | Timber for the market, construction, furniture, part of the sacred forest, leaf parts locally used to treat some infections | Ig | 007 |
| 54 | *Justicia schimperiana (Hochst. ex Nees)* | Acanthaceae | Gl, Hg | 2108 m, 8.60025 N, 37.881603 E | All | Live fence, control soil erosion | Ig |  |
| 55 | *Lathyrus sativus*^a^ L. | Fabaceae | Cl, Hg | 2087 m, 8.591932 N, 37.930255 E | MW | Legume crop that can be added to various traditional dishes like stew, soup | Id | 058 |
| 56 | *Lens culinaris* Medikus | Fabaceae | Cl, Hg | 2086 m, 8.591934 N, 37.930256 E | Ad, WT, AQ, SL, DGo, DG | Legume crop that can be added to various traditional dishes like stew, soup | Id | 060 |
| 57 | *Linum usitatissimum* L. | Linaceae | Cl, Hg | 3290 m, 8.802135 N, 37.862449 E | All | Oilseed, boiled uncrushed seeds are drunk to treat a stomachache | Ig | 002 |
| 58 | *Lycopersicon esculentum*^a^ Mill. | *Solanaceae* | Hg | 1997 m, 8.58315 N, 37.84197 E | MW, DGo, DG | Vegetable used in salads and spicy salsas | Id | 054 |
| 59 | *Maesa lanceolata* Forssk. | Myrsinaceae | Fp, Gl, Hg | 2302 m, 8.657146 N, 37.904454 E | SL | Live fence, pounded leaves used for treating skin infections, control soil erosion | Ig | 050 |
| 60 | *Malus domestica Borkh* | Rosaceae | Hg | 3073 m, 8.46928 N, 37^.^52558 E | HW | Tree crops, ripe edible fruit | Id | 008 |
| 61 | *Mangifera indica*^a^ L. | Anacardiaceae | Cl, Hg | 1997 m, 8.58315 N, 37.84197 E | DG | Tree crops, ripe edible fruit with delicious flesh | Id | 054 |
| 62 | *Maytenus arbutifolia* (A. Rich.) Wilczek | Celastraceae | Fp, Gl, Hg | 2302 m, 8.657146 N, 37.904454 E | SL | Live fence, flowers provide pollen and nectar for honeybees, firewood | Ed | 050 |
| 63 | *Millettia ferruginea* (Hochst.) Bak. | Fabaceae | Cl, Fp, Hg | 2305 m, 8.656886 N, 37.954046 E | DW, MW | Construction, coffee shade, roots used as a local medicine for eczema, flowers provide pollen and nectar for honeybees, soil fertility | Ed | 034 |
| 64 | *Morus alba L.* | Moraceae | Hg | 3073 m, 8^.^46.928 N, 37^.^52558 E | HW | Tree crops, ripe edible fruit | Id | 009 |
| 65 | *Musa x-paradisiaca*^a^ L. | Musaceae | Hg | 1997 m, 8.58315 N, 37.84197 E | DG | Tree-like perennial crop, used for food and commercial purposes | Ig | 054 |
| 66 | *Myrica salicifolia* A. Rich. | Myricaceae | All | 3215 m, 8.803247 N, 37.865236 E | HW, Ad, CS, AQ | Construction, furniture, falling leaves increase soil fertility, toothbrush | Ig | 004 |
| 67 | *Myrsine africana* L. | Myrsinaceae | Fp, Gl | 3029 m, 8.784141 N, 37.837693 E | Ad, CS, AQ | Fodder, firewood, fruits used as local medicine against stomach problem | Ig | 019 |
| 68 | *Myrsine melanophloeos ^b^* (L.) R. Br. | Myrsinaceae | Fp, Gl | 3042 m, 8.783743 N, 37.838192 E | Ad, CS | Construction, firewood, furniture | Ig | 021 |
| 69 | *Ocimum lamiifolium* Hochst. ex. Benth | Lamiaceae | Fp, Gl, Hg | 2108 m, 8.60025 N, 37.881603 E | All | Widely used as a local medicine to relieve fever and febrile illness | Ig | 024 |
| 70 | *Olea europaea* L. subsp. cuspidate | Oleaceae | All | 3027 m, 8.784444 N, 37.83709 E | HW, Ad, CS, AQ, WT | House construction, firewood, charcoal, furniture, used to fumigate pots for milk and local beverage, sticks for toothbrushes, part of the sacred forest | Ig | 018 |
| 71 | *Olinia rochetiana* A. Juss. | Oliniaceae | Fp, Gl | 3029 m, 8.784141 N, 37.837693 E | Ad, CS, AQ | Construction, firewood, farm tools | Ig | 019 |
| 72 | *Osyris quadripartita* Decn. | Santalaceae | Fp, Gl | 3042 m, 8.783696 N, 37.838218 E | Ad | Fodder, firewood, erosion control in a sloped area | Ig | 020 |
| 73 | *Persea americana*^a^ Mill. | Lauraceae | Hg | 2267 m, 8.65356 N, 37.907198 E | SL | Tree crop, fruits used as a source of protein and fat and for sale in local markets | Id | 047 |
| 74 | *Pisum sativum* L. | Fabaceae | Cl, Hg | 2087 m, 8.591932 N, 37.930255 E | Ad, WT, DW, MW | Legume crop that can be added to various traditional dishes like stew, soup | Id | 059 |
| 75 | *Plectranthus edulis*^a^ (Vatke) Agnew | Lamiaceae | Cl, Hg | 2311 m, 8.652808 N, 37.9045541 E | WT | Vegetable, cultivated for its edible tubers | Ig | 031 |
| 76 | *Podocarpus falcatus* (Thunb.) Mirb. | Podocarpaceae | All | 2896 m, 8.754183 N, 37.913359 E | AQ, DW, SL, MW | Construction, part of the sacred forest, used for protecting soil from erosion, firewood | Ig | 033 |
| 77 | *Prunus africana* (Hook.f.) Kalkm. | Rosaceae | Fp, Gl | 2267 m, 8.65356 N, 37.907198 E | AQ, SL | Timber tree, coffee shade, construction, furniture, stem barks used against urinary disorders, control erosion, firewood | Ig | 052 |
| 78 | *Prunus persica L.),* | Rosaceae | Hg | 2311 m, 8.652808 N, 37.9045541 E | WT | Tree crops, ripe edible fruit | Id | 031 |
| 79 | *Psidium guajava*^a^ L. | Myrtaceae | Cl, Hg | 1997 m, 8.58315 N, 37.84197 E | DG | Tree crop, ripe edible fruit | Id | 054 |
| 80 | *Pterolobium stellatum* (Forssk.) Brenan | Fabaceae | Fp, Gl, Hg | 2302 m, 8.657146 N, 37.904454 E | SL | Live fence, protection of soil from erosion | Ig | 050 |
| 81 | *Rhamnus prinoides* L' Herit | Rhamnaceae | Hg | 2896 m, 8.754183 N, 37.913359 E | Ad | Tree crop, used for making local alcoholic beverages | Ig | 033 |
| 82 | *Rhus glutinosa* A. Rich. Subsp. *neoglutinosa* | Anacardiaceae | Cl, Fp, Gl | 2333 m, 8.652762 N, 37.904476 E | SL, DG | Construction, farm tools, firewood, control erosion, forage for goats | Ed | 047 |
| 83 | *Ricinus communis* L. | Euphorbiaceae | Gl, Hg, | 2333 m, 8.652762 N, 37.904476 E | SL | Oilseed serves as lubrication, herbal medicine for wounds, and spices | Ig | 046 |
| 84 | *Rosa abyssinica* Lindl. | Rosaceae | Fp, GL, Hg | 3290 m, 8.802135 N, 37.862449 E | All | Edible fruit, live fence | Ig | 002 |
| 85 | *Rubus apetalus* Poir. | Rosaceae | Fp, Gl | 3027 m, 8.784444 N, 37.83709 E | Ad, CS, WT, AQ | Edible fruit, live fence, flowers provide pollen and nectar for honeybees | Ig | 016 |
| 86 | *Rumex nervosus* Vahl | Polygonaceae | Fp, GL, Hg | 3215 m, 8.803247 N, 37.865236 E | All | Firewood, fodder, children eat a pulled young shoot part | Ig | 004 |
| 87 | *Saccharum officinarum*^a^ L. | *Poaceae* | Hg | 1997 m, 8.58315 N, 37.84197 E | DW | Green stem used for making juice and crystallization sugar | Id | 054 |
| 88 | *Sesbania sesban* (L.) Merr. | Fabaceae | Fp, Gl, Hg | 1997 m, 8.58315 N, 37.84197 E | MW, DG | Shade for young coffee plants, forage for domestic animals, control erosion, construction, firewood | Ig | 054 |
| 89 | *Solanecio gigas* (Vatke) C. Jeffrey | Asteraceae | Fp, Hg | 2911 m, 8.754148 N, 37.913583 E | CS, AQ, | Live fence, flowers provide pollen and nectar for honeybees | Ed | 029 |
| 90 | *Solanum anguivi* Lam. | Solanaceae | Fp, Gl | 2267 m, 8.65356 N, 37.907198 E | CS | Chewing a clean root for treating bloating of the stomach locally | Ig | 046 |
| 91 | *Solanum tuberosum* L. | *Solanaceae* | Cl, Hg | 2911 m, 8.754148 N, 37.913583 E | All | Vegetable, major staple foods and used in a wide range of dishes including boiled, baked, roasted, and used in stew preparation | Ig | 030 |
| 92 | *Sorghum bicolor*^a^ L. | Poaceae | Cl, Hg | 2305 m, 8.656886 N, 37.954046 E | DW | Cereal crop, grown for forage and seeds used for making injera and local beverage | Ig | 035 |
| 93 | *Spathodea campanulata* P. Beauv. | Bignoniaceae | Hg, Gl | 2087 m, 8.591932 N, 37.930255 E | DG | Shade for coffee, street tree, control erosion, firewood | Ig | 054 |
| 94 | *Syzygium guineense* (Wild.) DC. subsp. *afromontanum* | Myrtaceae | Cl, Fp, Gl | 2333 m, 8.652762 N, 37.904476 E | WT, SL | Edible fruit, construction, firewood, furniture, control soil erosion | Ig | 45 |
| 95 | *Syzygium guineense* (Wild.) DC. subsp. *guineense^a^* | Myrtaceae | Fp, Gl | 2267 m, 8.65356 N, 37.907198 E | SL | Edible fruit, part of riparian vegetation and maintain streams, construction, firewood, furniture, control soil erosion | Ig | 053 |
| 96 | *Triticum aestivum* L. | Poaceae | Cl, Hg | 2087 m, 8.591932 N, 37.930255 E | All | Cereal crop that is used for roasted grain and bread | Ig | 057 |
| 97 | *Triticum durum* Desf. | Poaceae | Cl, Hg | 2911 m, 8.754148 N, 37.913583 E | AQ | Cereal crop that is used for roasted grain and porridge | Ig | 029 |
| 98 | *Vernonia amygdalina* Del. | Asteraceae | Fp, Gl, Hg | 2087 m, 8.591932 N, 37.930255 E | DG | Leaves and barks used as local medicines to treat many diseases, fodder, flowers attract honeybees, live fence, falling leaves enhance soil fertility, firewood | Ig | 047 |
| 99 | *Vernonia auriculifera* Hiern. | Asteraceae | All | 2108 m, 8.60025 N, 37.881603 E | DGo, DG | Firewood, fodder, serves as a borderline between two farmers of croplands | Ig | 056 |
| 100 | *Vicia faba* L. | Fabaceae | Cl, Hg | 2086 m, 8.591934 N, 37.930256 E | HW, Ad, CS, WT, AQ, DGo | Legume crop with beans that can be added to various traditional dishes like stew, soup | Id | 059 |
| 101 | *Zea mays*^a^ L. | Poaceae | Cl, Hg | 2305 m, 8.656886 N, 37.954046 E | DW, SL, MW, DGo | Cereal crop, grown for forage and seeds used in various traditional dishes | Id | 035 |
| 102 | *Vachellia abyssinica* Hochst. | Fabaceae | All | 3027 m, 8.784444 N, 37.83709 E | All | Firewood, charcoal, live fence, shade for livestock, maintain streams, nitrogen fixation, and soil fertility | Ig | 017 |
| 103 | *Vachellia seyal*^a^ Del. | Fabaceae | Fp, Gl, Hg | 2087 m, 8.591932 N, 37.930255 E | DG | Live fence, leaves are forage for browsers, soil fertility, shade for cattle | Ig | 047 |
