# Supplementary material for: Land use and land cover dynamics and traditional agroforestry practices in Wonchi District, Ethiopia
Source: PeerJ. 2022 Feb 22;10:e12898. doi: 10.7717/peerj.12898 (PMC8877395; doi:10.7717/peerj.12898)
Supplement: Supplemental Information 5 [file peerj-10-12898-s005.docx]

| LULC Class | 1985 | | 2001 | | 2019 | |
| --- | --- | --- | --- | --- | --- | --- |
|  | Area (ha) | Area (%) | Area (ha) | Area (%) | Area (ha) | Area (%) |
| Agroforestry cover | 14553.4 | 31.1 | 14669.0 | 31.4 | 16305 | 34.9 |
| Cropland | 18081.5 | 38.7 | 16636.3 | 35.6 | 11571.3 | 24.8 |
| Forest cover | 4396.8 | 9.4 | 4031.2 | 8.6 | 877.8 | 1.9 |
| Settlement and roads | 5831.2 | 12.5 | 5299.5 | 11.3 | 14752.6 | 31.6 |
| Shrub cover | 3307.9 | 7.1 | 5595.2 | 12.0 | 2794.2 | 6.0 |
| Water body | 565.5 | 1.2 | 505.1 | 1.1 | 435.42 | 0.9 |
| Total | 46736.4 | 100.0 | 46736.4 | 100.0 | 46736.4 | 100.0 |
