# Supplementary material for: Land use and land cover dynamics and traditional agroforestry practices in Wonchi District, Ethiopia
Source: PeerJ. 2022 Feb 22;10:e12898. doi: 10.7717/peerj.12898 (PMC8877395; doi:10.7717/peerj.12898)
Supplement: Supplemental Information 6 [file peerj-10-12898-s006.docx]

| Variables for the source of income, the use, and status of natural resources | Frequency (N = 100) |
| --- | --- |
|  |  |
| I. The main sources of income in your household (more than one option is possible). |  |
| 1. Crop farming | 98 |
| 1. Livestock | 98 |
| 1. Bee farming | 77 |
| 1. Others (such as labor, fuel sale, employee and renting the land) | 26 |
| II. Types of natural resources that are found in your community (more than one option is possible.) |  |
| 1. Natural forest (private, public, sacred, riparian forest) | 68 |
| 1. Plantation of agroforestry trees (exotic, indigenous, or mixed) | 97 |
| 1. Shrubs (open and protected/enclosed) and grasslands | 82 |
| 1. Crops in the farmlands and homegardens | 100 |
| 1. Water bodies (permanent streams, rivers, lake) and swamps | 71 |
| III. Have you noticed any visible changes in the LULC patterns over the last 34 years? |  |
| 1. Yes | 97 |
| 1. No | 3 |
| 1V. If yes, what kind of prevailing LULC changes have you observed over the last 34 years? (more than one option is possible.) |  |
| 1. Forest cover to settlement and roads | 67 |
| 1. Forest cover to agroforestry cover | 71 |
| 1. Water body and swamps to grassland and shrub cover | 12 |
| 1. Cropland to agroforestry cover | 55 |
| 1. Cropland to shrub cover | 16 |
| \| **V. What is the current status of vegetation in the area in comparison to the last 34 years?** \| \| --- \| |  |
| 1. Still intact/no visible change | 2 |
| 1. Slightly disturbed | 76 |
| 1. Heavily disturbed/ cleared | 22 |
| VI. If your answer is B or C on QV, what are the major driving causes of vegetation depletion or LULCs? (More than one option is possible.) |  |
| 1. Expansion of agriculture | 88 |
| 1. Expansion of human settlement and developmental construction | 69 |
| 1. Charcoal and firewood | 49 |
| D. Livestock grazing | 45 |
| VII. What are the effects of LULC on natural plant resources? |  |
| A. Positive effects | 6 |
| B. Negative effects | 94 |
| In which governmental regime has the gradual negative changes been observed? |  |
| 1. The Emperor H/Selassie regime | 3 |
| 1. The Derg regime | 20 |
| 1. The EFDR regime | 77 |
| VIII. Have there been changes shown in the types of crops that you have grown on your farmland in the last 34 years? |  |
| 1. Yes | 34 |
| 1. No | 66 |
| IX. Do you have any kind of agroforestry practice to mitigate climate change (microclimate) and conserve natural resources including plant and soil resources? |  |
| 1. Yes | 94 |
| 1. No | 6 |
