# Supplementary material for: Land use and land cover dynamics and traditional agroforestry practices in Wonchi District, Ethiopia
Source: PeerJ. 2022 Feb 22;10:e12898. doi: 10.7717/peerj.12898 (PMC8877395; doi:10.7717/peerj.12898)
Supplement: Supplemental Information 7 [file peerj-10-12898-s007.docx]

| Research site | Main source of income | | | | Agro-ecological zone type |
| --- | --- | --- | --- | --- | --- |
|  | Crop-livestock production | Crop-livestock-beekeeping | Crop-livestock-beekeeping, and local trade/shopping | Other |  |
| Harro Wonchi | 4 | 4 |  | 2 | Highland |
| Adofa | 2 | 6 | 2 |  |  |
| Chebose Seleten | 5 | 5 |  |  |  |
| Wendo Talfe | 10 |  |  |  |  |
| Azer Qerensa |  | 10 |  |  |  |
| Dae Wandimtu |  | 10 |  |  | Midland |
| Sonko Lekake |  | 10 |  |  |  |
| Miti Walga |  | 1 | 9 |  |  |
| Degoye Galle |  |  | 10 |  |  |
| Dimtu Goditi |  | 10 |  |  |  |
| Total frequency | 21 | 56 | 21 | 2 | 100 |
